# P01.10. Effects of caloric restriction combined with traditional chinese phytomedicine on the glucolipid metabolism in Wistar Rats with insulin resistance

**DOI:** 10.1186/1472-6882-12-S1-P10

**Published:** 2012-06-12

**Authors:** C Li, S Li, A Michalsen, J Qin

**Affiliations:** 1 Charité, University Medical Centre Berlin, Berlin, Germany; 2 The First Affiliated Hospital of Sun Yat-Sen University, Guangzhou, China

## Purpose

Insulin resistance (IR) and hyperlipemia are two important pathophysiological manifestations in metabolic diseases. This research aims to investigate the influence of complete caloric restriction (CR) combined with Chinese medicine decoction (ling-gui-zhu-gan,LGZG) on glucose and lipid metabolism in rats with IR.

## Methods

48 male Wistar rats were randomly assigned by 3:1 ratio to a high-fat diet group, which was fed with a high-fat diet for 12 weeks, and a control group which received a standard diet, respectively. Rats in the high-fat diet group were randomized by a ratio of 1:1:1 into a CR group, a CR+LGZG group that additionally received a LGZG decoction, and a group which continued the high-fat diet. Rats in the CR+LGZG group were administered intragastrically LGZG decoction daily, and the other two groups received 3ml of saline. Outcomes were assessed at baseline, at the end of the 12^th^ week, and after the 3-day CR.

## Results

At the end of the 12^th^ week, the rats with insulin resistance were established successfully in the high-fat diet group. After CR, fasting plasma glucose (FPG), fasting insulin (FINS) and insulin resistance index (IRI) were reduced by 10.0% (p>0.05), 61.6% (p<0.01) and 65.5% (p<0.01) compared with standard diet, while CR+LGZG led to reductions of 43.9% (p<0.01), 73.4% (p<0.01) and 85.9% (p<0.01). Compared with CR only, CR+LGZG led to significant decreases in FPG (p<0.01), FINS (p<0.05) and IRI (p<0.01). Blood lipids (CHO, TG, HDL-C, LDL-C) were significantly reduced in both CR only and CR+LGZG compared with standard diet (p<0.01), while there was no difference between CR with or without LGZG.

## Conclusion

CR+LGZG decoction has greater effects on glucose metabolism than only CR. Both CR and CR +LGZG improve lipids to the same extent. The beneficial effect of an additional herbal medication during CR might be related to fasting-induced gluconeogenesis. Human studies on the beneficial impact of CR+Chinese medicine on diabetic patients are warranted.

